# Antibiotic prophylaxis in breast cancer surgery. A randomized controlled trial^1^

**DOI:** 10.1590/s0102-865020200090000007

**Published:** 2020-09-30

**Authors:** Rubens Murilo de Athayde Prudencio, Fabíola Soares Moreira Campos, Ana Beatriz Alkmim Teixeira Loyola, Ivanildo Archangelo, Neil Ferreira Novo, Lydia Masako Ferreira, Daniela Francescato Veiga

**Affiliations:** I Master, Professional Master’s Program in Applied Health Sciences, Universidade do Vale do Sapucaí (UNIVÁS), Pouso Alegre-MG, and Breast Cancer Unit, Hospital São Cristóvão, Sao Paulo-SP, Brazil. Conception, design, intellectual and scientific content of the study; acquisition, interpretation and analysis of data; manuscript writing; final approval.; II Master, Professional Master’s Program in Applied Health Sciences, and Hospital das Clínicas Samuel Libânio, UNIVÁS, Pouso Alegre-MG, Brazil. Conception, design, intellectual and scientific content of the study; acquisition, interpretation and analysis of data; manuscript writing; final approval.; III PhD, Associate Professor, Professional Master’s Program in Applied Health Sciences, UNIVÁS, Pouso Alegre-MG, Brazil. Conception, design, intellectual and scientific content of the study; interpretation and analysis of data; manuscript writing; critical revision; final approval.; IV MD, Department of Breast Surgery, UNIVÁS, Pouso Alegre-MG, Brazil. Conception, design, intellectual and scientific content of the study; acquisition of data; final approval.; V PhD, Full Professor, Department of Biostatistics, UNIVÁS, Pouso Alegre-MG, Brazil. Interpretation of data, statistical analysis, critical revision, final approval.; VI PhD, Chairwoman, Head, Postgraduate Program in Translational Surgery, Division of Plastic Surgery, Universidade Federal de São Paulo (UNIFESP), Sao Paulo-SP, Brazil. Critical revision, final approval.; VII PhD, Associate Professor, Professional Master’s Program in Applied Health Sciences, UNIVÁS, Pouso Alegre-MG, and Postgraduate Program in Translational Surgery, UNIFESP, Sao Paulo-SP, Brazil. Conception, design, intellectual and scientific content of the study; interpretation and analysis of data; manuscript writing; critical revision; final approval.

**Keywords:** Antibiotic Prophylaxis, Breast Neoplasms, Postoperative Care, Surgical Wound Infection

## Abstract

**Purpose:**

To assess the effect of antibiotic prophylaxis on surgical site infection (SSI) rates in women undergoing breast cancer surgery in two tertiary hospitals in Brazil.

**Methods:**

This was a randomized, double-blind, placebo-controlled, parallel-group clinical trial. A total of 124 women without independent risk factors for SSI were randomly assigned to receive either cefazolin (antibiotic group, n = 62) or placebo (control group, n = 62) as preoperative prophylaxis. After surgery, all surgical wounds were examined once a week, for four weeks, according to the Centers for Disease Control and Prevention definitions and classifications for SSI.

**Results:**

Baseline characteristics were homogeneous between the two groups. Only one patient in the antibiotic group developed SSI, which was classified as superficial incisional. The overall SSI rate was low, with no significant difference between groups.

**Conclusion:**

Antibiotic prophylaxis had no significant effect on reducing SSI rates in women without independent risk factors for SSI undergoing breast cancer surgery.

## Introduction

Surgical site infections (SSI) account for 14-16% of all nosocomial infections in inpatients and are the most common infections among surgical patients^1^. Despite being considered a clean surgical procedure, breast cancer surgery has been associated with high SSI rates, ranging from 3% to 15%^2,3^. Prevention of SSI is of paramount importance due to its significant impact on patient morbidity and health care costs.

The development of SSI is associated with prolonged hospitalizations, increased hospital costs, increased risk of intensive care unit admission, high rates of hospital readmissions, and increased risk of death^2,4^. This issue is particularly important for patients undergoing breast cancer surgery, because the presence of an infected breast wound may delay the start of adjuvant therapy, which may negatively affect local control and survival^5,6^.

Antibiotic prophylaxis is routinely used in breast cancer surgery, especially when drainage or breast reconstruction is performed, despite the lack of evidence of its efficacy^7-9^. However, adverse reactions may occur and drug resistance has also to be considered^2,10^. In addition, the use of suboptimal dosing of prophylactic antibiotics is an independent risk factor for SSI in breast surgery^6,10^.

Evidence supports the administration of preoperative antimicrobial agents when the incision is made only when its indication is based on clinical practice guidelines specific to the procedure being performed^11^. However, there is no consensus in the literature on the use of prophylactic antibiotics in breast cancer surgery^7,9,10^.

This trial was designed to compare SSI rates associated with the use of either antibiotic prophylaxis or placebo in patients undergoing breast cancer surgery. The aim of the study was to test the hypothesis that prophylactic antibiotic administration would not affect SSI rates.

## Methods

The study was approved by the Research Ethics Committee of UNIVÁS (approval number 433.590) and performed in accordance with the Resolution 466/12 of the Brazilian National Health Council (CNS) on research involving human beings and with the ethical principles of the1964 Declaration of Helsinki and its subsequent amendments. The trial was registered at ClinicalTrials.gov, number NCT02809729. Written informed consent was obtained from all patients before their inclusion in the study, and anonymity was assured.

### Settings and locations

The trial was conducted at the Hospital São Cristóvão and Hospital São Rafael, both located in Sao Paulo-SP, and affiliated with the Universidade do Vale do Sapucaí (UNIVÁS). Patients were recruited in the breast outpatient clinics and operated on Hospital São Cristóvão and Hospital São Rafael.

### Eligibility criteria

This is a randomized, double-blind, placebo-controlled, parallel-group clinical trial. Patients were recruited between April 2015 and December 2016, and were followed-up from May 2015 to January 2017. Eligible participants were all women aged 20 to 75 years, with a diagnosis of breast cancer, who were referred for surgical treatment. Exclusion criteria were body mass index (BMI) > 30 kg/m^2^, prior neoadjuvant chemotherapy, be a candidate for immediate breast reconstruction, diabetes mellitus with a level of glycosylated hemoglobin ≥ 7%, American Society of Anesthesiologists (ASA) III score or higher, and failure to attend any of the weekly follow-up visits. Patients who required antibiotic therapy due to other clinical complications (e.g., cystitis, pneumonia, among others) were also excluded from the study.

### Randomization and blinding

Of 201 recruited patients, 124 met inclusion criteria and were randomly assigned to the antibiotic group (n=62) or control group (n=62) based on a computer-generated randomization chart with a 1:1 allocation ratio (Bioestat 5.3, Instituto Mamirauá, Pará, Brazil).

The allocation sequence was concealed from patients, physicians, investigators, and outcome assessors. A pharmacist, who held the allocation sequence, prepared the vials on the day of the operation. An opaque, sealed envelope was attached to each vial, containing information regarding the vial content.

### Outcomes

The primary outcome was SSI rates. SSI Surveillance was performed prospectively via weekly follow-up visits held in the participating outpatient clinics. After surgery, all surgical wounds were examined once a week, for four weeks, by the surgeon and surgeon assistants, according to the Centers for Disease Control and Prevention (CDC) definitions and classifications for SSI surveillance^12^. Surgical site infections were classified as superficial incisional, deep incisional, or organ/space infections, and defined as infections occurring within 30 days after the operation^12^.

### Interventions

As preoperative prophylaxis, the antibiotic group received 2 g of cefazolin in 100 ml of saline and the control group received 100 ml of 0.9% saline (placebo). The cefazolin and placebo solutions were identical in appearance and supplied in numbered identical vials.

The sealed envelope containing information regarding the vial content was opened by the anesthesiologist at the time of the procedure, who needed to know the vial content in case of antibiotic-associated adverse reactions. The solution was administered intravenously 30 minutes before induction of anesthesia.

Between 30 minutes and 1 hour before being transferred to the surgical unit, the patients were instructed to shower with 4% chlorhexidine gluconate^14^. After induction of anesthesia, skin antisepsis was performed using 4% chlorhexidine gluconate solution, which was subsequently removed with sterile gauzes, followed by application of 0.5% chlorhexidine-alcohol solution^15^. All procedures were performed by the same surgeon and two assistant surgeons. Immediately after the procedure, the surgical wound site was covered with a sterile dressing, which was changed prior to the patient’s discharge. The patients were instructed to maintain the dressing for 48 hours, after which the dressing should be removed, and the wound should be washed daily with warm water and neutral soap during shower, and covered with a dry sterile gauze dressing.

The sealed envelope containing information regarding the vial content was opened by the anesthesiologist at the time of the procedure, who needed to know the vial content in case of antibiotic-associated adverse reactions. The solution was administered intravenously 30 minutes before induction of anesthesia.

### Implementation

The generation of the allocation sequence was performed by the senior author, who did not participate in the recruitment of patients or assessment of the outcome. Two authors, members of the surgical team, enrolled participants, and an anesthesiologist assigned participants to interventions.

### Statistical analysis

The sample size was calculated based on a previous comparative study with 100 patients^11^. SSI rate was chosen as the primary outcome measure. For a two-sided significance level of 5% and a power of 80%, the sample size of 62 patients in each group would be required to detect a significant difference in SSI rates between the antibiotic and control groups.

Categorical variables were expressed as frequencies and percentages, while quantitative variables were expressed as median and interquartile range or mean and standard deviation (SD). The Mann-Whitney test was used to determine differences between groups regarding age, BMI, and operating time. Fisher’s exact test was used to compare SSI rates between groups.

Data were analyzed using Bioestat, version 5.3 (Instituto Mamirauá, Pará, Brazil). All statistical tests were performed at a significance level α of 0.05 (p<0.05).

## Results

A total of 124 patients were included in the study. Most of them were white (95.2%) and postmenopausal women (83.9%). Baseline demographic characteristics were homogeneous between groups, as well as types of surgical procedures and operating time (Table 1).

**Table 1:** Patient characteristics in the antibiotic and control groups.

Characteristic	Antibiotic (n = 62)	Control (n = 62)	p-value*
Age, years, median (IQR)	62.2 (36-75)	63.4 (23-75)	0.500
BMI, kg/m^2^, median (IQR)	26.3 (21-30)	27.3 (21-30)	**0.016**
Smoking, N (%)	14 (22.6)	6 (9.7)	**0.014**
Mastectomy, N (%)	7 (11.3)	6 (9.7)	1.000
Quadrantectomy, N (%)	49 (79.0)	43 (69.3)	0.111
Segmentectomy, N (%)	6 (9.7)	13 (21.0)	**0.049**
Sentinel lymph-node biopsy, N (%)	52 (83.9)	56 (90.3)	0.293
Axillary dissection, N (%)	14 (22.6)	12 (19.3)	0.494
Operating time, min, median (IQR)	65 (45-120)	67 (30-120)	0.355

BMI, body mass index; IQR, interquartile range; N, population size; n, sample size.

Numbers in bold indicate statistical significance.

*p-values for age, BMI, and operating time were calculated using the Mann-Whitney test; all other p-values were determined using Fisher’s exact test.

**Figure 1:**
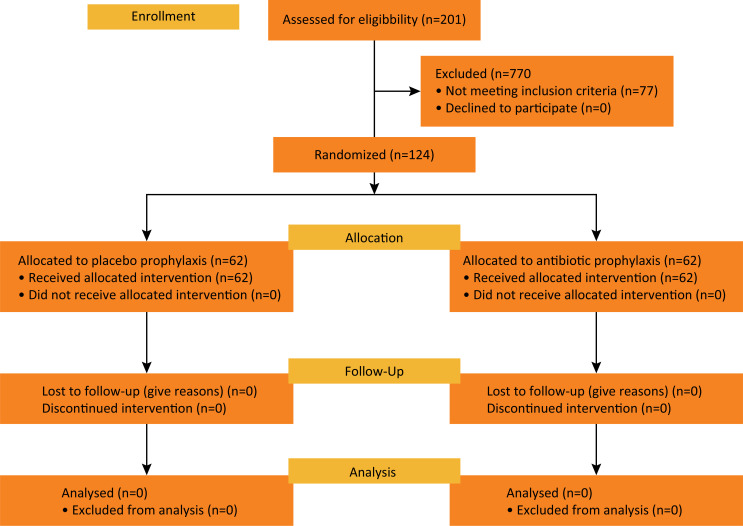
Flow diagram of study participants.

All 124 patients completed the trial and were included in the data analysis. Figure 1 shows the flow of study participants^16^. Drains were used in all patients, with a median duration of 5.7 days (antibiotic group, 5.8 days; control group, 6.0 days; p = 0.55). Postoperative complications occurred in 15 (24.2%) patients in the antibiotic group and in 16 (25.8%) patients in the control group (p = 0.83). The complications included hyperemia with local swelling (25.0%), serous fluid discharge (19.0%), hematoma (13.0%), hematic discharge (13.0%), small granulomas (13.0%), seroma formation under the scar (13.0%), and focal dehiscence (6.0%).

Only one patient in the antibiotic group developed SSI. The infection was detected in the first postoperative week and was classified as a superficial incisional SSI. As it was considered a rare event, the Fisher exact test was applied to compare the groups. The overall SSI rate was 0.8%, with no difference between groups (p = 0.50).

## Discussion

We hypothesized that antibiotic prophylaxis administration would not influence postoperative SSI rates in women undergoing breast cancer surgery and the study results confirmed this hypothesis. In 2017, the CDC published updated guidelines for the prevention of SSI, based on an extensive literature review^12^. With an evidence category IB (strong recommendation), these guidelines state that preoperative antimicrobial prophylaxis should be administered only when indicated, based on published clinical practice guidelines^12^. The issue remains controversial as to the use of prophylactic antibiotics in breast surgery.

A Cochrane systematic review indicated that the use of prophylactic antibiotics preoperatively significantly reduced the incidence of SSI in patients undergoing surgery for breast cancer without immediate reconstruction^2^. Other studies, however, showed no difference in SSI rates with the use (or not) of preoperative antibiotic prophylaxis in breast cancer surgery^3,9,10^, similar to the results of the present study. Another study even observed a higher rate of SSI in patients who received antibiotic prophylaxis^10^. The authors conducted a retrospective study of patients undergoing non-reconstructive breast surgery in which 454 patients received a single dose of preoperative antibiotic and 401 received no antibiotic prophylaxis, and found a significantly higher SSI rate (12%) in the antibiotic group compared to that (4%) in the non-antibiotic group (p<0.0001). This difference was attributed to a possible underdosing of antibiotics, which was associated with higher rates of SSI^10^.

The CDC guidelines recommend that, for clean and clean-contaminated procedures, prophylactic antibiotics should not be administered after the surgical incision is closed, even in the presence of a drain^12^. However, these guidelines are not specific to breast surgery. A retrospective study of 425 patients undergoing mastectomy with drain use found a higher rate of SSI among patients who did not receive prophylactic antibiotics postoperatively^8^.

In our institutions, the Hospital Infection Control Committee recommends intravenous administration of 2g of cefazolin as preoperative prophylaxis in mastectomy and breast-conserving surgery (quadrantectomy and segmentectomy), with or without sentinel lymph-node biopsy. Cefazolin (a first-generation cephalosporin) is one of the most commonly used prophylactic antibiotics in patients undergoing breast cancer surgery^2,9,10^.

The present study had an overall SSI rate of only 0.8%, meaning that only one patient developed SSI within 30 days of surgery. It should be noted that this single case occurred in the antibiotic group. This low SSI rate, different from those reported in the literature, maybe attributed to some factors. One of these factors refers to the eligibility criteria, as patients with known independent risk factors for SSI, such as obesity, prior neoadjuvant chemotherapy, be a candidate for immediate breast reconstruction, and presence of comorbidities were not included in the study^4,10,17^. In addition, preoperative SSI prevention measures were standardized and strictly followed, including preoperative shower with antiseptic agent and antisepsis^14,15^. Another relevant factor is the use of dressings, which is either not mentioned or performed empirically in most studies. The CDC recommends that closed incisions be covered with a sterile dressing, which should be applied immediately after surgery and maintained for 24 to 48 hours^18^. In the present study, the wound dressing was changed before patient discharge, and all patients were instructed to remove the dressing after 48 hours and then take daily showers with an uncovered surgical wound.

A major limitation of this study is the use of strict eligibility criteria, which may prevent generalization of the results. Thus, the results apply to women without independent risk factors for SSI undergoing breast cancer surgery rather than to candidates for breast cancer surgery in general. Another important limitation refers to the power of the study. Sample size calculation was based on a study on reduction mammaplasty^15^, which was chosen because its eligibility criteria (for a group of patients undergoing major breast surgery of higher complexity than that of mastectomy without reconstruction) was similar to those used in the present study. However, this may have led to an underestimation of the sample size. Future studies, with larger samples, are mandatory to fully elucidate the issue and establish guidelines for clinical practice. Statistical differences in BMI and smoking between groups may also be considered as possible causes of bias. When a randomization is performed, the allocation is by chance. The researchers are not able to select homogeneous groups; what they can do is to establish strict eligibility criteria. Thus, due to randomization, eventual differences should be attributed to chance. The use of a strict methodology and randomization of patients reduced this bias effect, which does not seem to have affected the final results. In fact, although the groups differed in relation to variables known as risk factors for SSI (higher BMI and smoking), there was no difference in relation to SSI. For decades, the use of antibiotic prophylaxis in clean surgical procedures has been considered an essential part of surgical practice protocols in many institutions. There is strong evidence of the benefits of administering a dose of prophylactic antibiotics before induction of anesthesia, as performed in the present study, but only when indication is based on published clinical practice guidelines^11^. However, at present, no such guidelines exist for breast cancer surgery. In this respect, the present findings provide further support for ongoing efforts to establish standards for the use of preoperative antibiotic in breast cancer surgery.

A recent systematic review indicated that preoperative administration of prophylactic antibiotics may reduce infection rates among patients undergoing breast cancer surgery^19^. However, recent works have highlighted that further studies are necessary to establish guidelines and protocols for clinical practice^19-21^.

Our results suggest that preoperative antibiotic prophylaxis is not effective in reducing SSI rates in women undergoing breast cancer surgery. Based on the low SSI rate observed in the present study and the lack of conclusive studies demonstrating its benefit, the authors believe that preoperative antibiotic prophylaxis may not be required in clean, elective breast cancer surgery. This practice is in line with the importance of prudent use of antibiotics to reduce the development of antibiotic resistance. However, the limited power of the current study decreases its external validity. Further studies, with much larger sample sizes, are necessary to provide evidence for the clinical practice.

## Conclusion

This study suggested that preoperative antibiotic prophylaxis had no significant effect on reducing SSI rates in women without independent risk factors for SSI undergoing breast cancer surgery, thus suggesting that its routine use in this population may be unnecessary.
